# Medical tourism in ophthalmology: learning from past mistakes

**DOI:** 10.1038/s41433-026-04672-1

**Published:** 2026-07-03

**Authors:** Vidhi Naik, Rishi Ramessur, Yarrow Scantling-Birch

**Affiliations:** 1 Aberdeen Royal Infirmary, National Health Service (NHS) Grampian, Aberdeen, UK; 2 National Health Service (NHS) Borders, Melrose, UK; 3 School of Informatics, University of Edinburgh, Edinburgh, UK; 4 Centre for Eye Research Australia, Royal Victorian Eye and Ear Hospital, Melbourne, VIC Australia

**Keywords:** Health services, Eye diseases

The burden of medical tourism to the National Health Service (NHS) has been estimated to cost between £1,058 and £19,549 per patient [1]. Outward tourism has increased fivefold, with 348,000 United Kingdom (UK) residents travelling abroad for healthcare between 2010 and 2022 [2]. Professional bodies, including the British Association of Aesthetic Plastic Surgeons, have issued warnings about travelling abroad for any cosmetic or surgical procedure [3].

The true cost of medical tourism remains difficult to quantify due to poor reporting, with higher costs associated with prolonged admissions, revision surgery, and ongoing NHS follow-up. Major motivators for individuals seeking outward medical care include: the length of waiting lists, the appeal of “hospitality-style” surgical packages, and the expense of private healthcare in the UK [1]. Cosmetic and ophthalmic surgeries were reported to be amongst the top tourism-related post-operative complications following bariatric surgery [1].

Internationally, price variation in cataract surgery remains substantial. The average price for bilateral cataract surgery (historical prices referenced to 2014 US dollar), inclusive of consultation, practitioner fees, intraocular lens (IOL), and follow-up, varies significantly: Hungary ($3717.20), Türkiye ($4347.81), India ($4706.90), United States ($5,243.59) and UK ($7564.15) [4]. While differences in labour market, regulation, and healthcare financing partly explain this disparity, they do not fully account for the substantial price variation observed for identical products across geographical settings [5]. Commercial pricing strategies act as a major contributing factor to this price variation. Manufacturers, healthcare institutions, and IOL companies price to what each market will bear, producing differential pricing between high-income and low-income nations fuelling the growth of medical tourism. By contrast, vertically-integrated healthcare providers, such as the Aravind Eye Care System (India), counter these pressures through economies of scale, high-volume theatre lists and in-house IOL manufacturing [6]. Additional economic drivers that impact the discrepancy in cataract surgery cost are highlighted in Table 1.

**Table 1:** The key drivers of cost differences in cataract surgery globally.

Factor	Mechanism for cost difference	Example
Surgical technique	Manual techniques, such as manual small-incision cataract surgery (MSICS), avoid costly phacoemulsification equipment and associated consumables, resulting in reduced surgical costs.	For example, MSICS is widely used in Nepal and India, achieving comparable visual outcomes to routine phacoemulsification at substantially lower cost [19, 20].
Labour & personnel costs	The cost of affiliated staff to deliver theatre, anaesthetic and allied healthcare support differs across nations. A major component of surgical expense will scale with local wages and Gross Domestic Product (GDP) per capita. Countries with lower GDP per capita have proportionally lower labour costs for surgeons, nurses, anaesthesiologists, and support staff [21].	In the United States, supply and demand economics dictate the higher-than-average price of cataract surgery in locations like Connecticut and Alaska where there is a scarcity of ophthalmologists [22].
Healthcare system structure & efficiency	Vertical integration, standardised protocols, high-volume theatre lists, and streamlined patient flow lower per-case cost. This is further confounded by reimbursement policies and healthcare structures, which can bridge between public and private sectors.	Aravind Eye Care System operate a highly efficient system, delivering on low-cost cataract surgery and save on IOL costs through in-house manufacturing [6].
Regulatory and compliance costs	Compliance, malpractice insurance, and administrative overhead inflate costs in heavily regulated systems, especially in Western Nations. Surgical indemnity for refractive and cosmetic eye surgery is one with the highest-premium.	India, Türkiye and Hungary benefit from fewer regulatory hurdles, unlike Western nations where the cost of insurance and administration reflect the cost of the surgical fees [23].

Global price differentials have not gone unexploited. Patients will travel across borders in search of affordable healthcare. Mobile health facilities have been heralded as imaginative solutions for filling gaps in healthcare access, an audacious example being the *Floks Floating Eye Clinic*, a converted passenger vessel managed by the Fyodorov Eye Surgery Network in the late Soviet to early post-Soviet era [7]. The aim was to deliver ophthalmic surgery in foreign ports, combining medical outreach with tourism. Presciently, it anticipated many of the tensions that now shape medical tourism: the attraction of affordable hospitality-based care and the challenges of long-term follow-up.

The *Peter I* commercial cruise liner was repurposed in the late 1980s with operating theatres, diagnostic laboratories, and patient accommodation. Crewed by ophthalmic teams affiliated with the Fyodorov Eye Microsurgery Complex (Moscow, Russia), it sailed to ports across the Mediterranean and the Middle East, offering a host of medical and surgical treatments, including radial keratotomies and cataract extraction [8]. Operations were only performed when the ship was berthed in port, and a range of luxurious leisure facilities complemented the medical services. The surgical throughput was high, reflecting Fyodorov’s characteristic assembly-line approach and contemporary surgical innovation [9].

Short-term outcomes appeared favourable, and visiting ophthalmologists praised the technical competence of the surgical teams [7]. However, the venture drew criticism. The European Union of Medical Specialists argued that a foreign-registered vessel conducting operations within territorial waters flouted local medical regulations and endangered the legal rights of European citizens [9, 10]. Critics also pointed to the lack of transparent outcome reporting and, most importantly, the lack of continuity of care. Patients who developed complications after departure relied on local healthcare systems, resulting in medicolegal uncertainty [7, 9].

The *Floks* experiment exposed the ambiguities of cross-border healthcare, fuelled by global price differentials. Was the ship accountable to its flag state, to the host nation’s regulations, or to neither? Ethically, it raised questions about the adequacy of informed consent when patients might not have appreciated the risks of disrupted follow-up.

Three decades on, the story resonates because its dilemmas persist. Ophthalmic tourism is expanding, particularly in refractive and oculoplastic surgery, with patients seeking more affordable options. Clinics in Türkiye, Spain, India, and Eastern Europe actively market procedures via social media, such as refractive surgery, keratopigmentation, and blepharoplasties to international patients; often bundled with high-quality hotel packages and sightseeing opportunities at a fraction of domestic costs [11, 12]. In some cases, intermediary agencies organise and book trips, raising ethical concerns regarding informed consent [13]. The parallels with Floks are striking. The same central question recurs: who provides follow-up when complications arise, and who incurs the associated costs? Reviews of medical tourism consistently note that the burden of follow-up lies with domestic healthcare systems, especially associated complications [1, 14]. A UK case series described complications following laser-assisted in situ keratomileusis (LASIK), IOL implantation, and cosmetic iris implants, with an estimated additional cost of £3,039 per patient complication [15].

The Royal College of Ophthalmologists has helped develop standards in cosmetic practice, yet it has not taken a decisive position on medical tourism in ophthalmology. While its guidance acknowledges the difficulties of providing aftercare for returning British patients and the risk of complications and emergencies, it offers little practical advice for patients considering treatment abroad, or for clinicians managing subsequent complications [16]. This lack of explicit direction represents a notable gap in professional guidance, despite the risk of long-term ocular morbidity following either intraocular and/or cosmetic complications [4, 17, 18].

Global price differentials in healthcare are driving patients in search of care unaffordable in their domestic nations. The experience of the Floks Floating Eye Clinic underscores a historical lesson for modern ophthalmic tourism: innovation in affordable healthcare must be matched by robust regulations that protect patient welfare. As demand grows for affordable healthcare and the medical tourism industry expands, the risks of fragmented follow-up, unclear liability, and absent outcome reporting become significant. The focus turns to making medical tourism safe by shared responsibility: regulators must clarify standards and accountability, providers must ensure continuity and transparency, and patients must exercise due diligence when seeking ophthalmic care abroad.

## Data Availability

Data sharing not applicable to this article as no datasets were generated or analysed during the current study.
